# Factors associated with Group A *Streptococcus emm *type diversification in a large urban setting in Brazil: a cross-sectional study

**DOI:** 10.1186/1471-2334-10-327

**Published:** 2010-11-11

**Authors:** Sara Y Tartof, Joice N Reis, Aurelio N Andrade, Regina T Ramos, Mitermayer G Reis, Lee W Riley

**Affiliations:** 1 University of California, Berkeley, School of Public Health, USA; 2 Faculdade de Farmácia, Universidade Federal da Bahia, Salvador, BA, Brazil; Gonçalo Moniz Research Centre, Oswaldo Cruz Foundation, Brazilian Ministry of Health, Salvador, BA, Brazil; 3 Hospital São Rafael-Monte Tabor, Salvador, BA, Brazil; 4 Department of Pediatrics, Federal University of Bahia School of Medicine, Salvador, BA, Brazil; 5 Gonçalo Moniz Research Centre, Oswaldo Cruz Foundation, Brazilian Ministry of Health, Salvador, BA, Brazil; 6 University of California, Berkeley, School of Public Health, USA

## Abstract

**Background:**

Group A *Streptococcus *(GAS) strain diversity varies across different regions of the world, according to low versus high-income countries. These differences may be related to geographic, environmental, socioeconomic, or host-related factors. However, local factors may also affect strain diversity. We compared the *emm *types of GAS isolates from children with and without sore throat in one large urban setting in Brazil.

**Methods:**

Children 3-15 years of age were consecutively recruited from slum and non-slum pediatric outpatient clinics between April-October, 2008. Throat cultures were performed and data intake forms were completed. GAS isolates were typed by *emm *sequencing.

**Results:**

From 2194 children, 254 (12%) GAS isolates were obtained. Of 238 GAS isolates that were *emm*-typed, 61 unique *emm *types were identified. Simpson's diversity index of the *emm *types was higher among isolates from slum children [97% (96%-98%)] than those of non-slum children [92% (89%-96%)]. Two *emm *types (66.0, 12.0) were more frequently isolated from children with sore throat (p < 0.05), and one *emm *type (27G.0) demonstrated a protective effect.

**Conclusions:**

The *emm *type diversity from children attending slum clinics resembled the *emm *diversity of low income countries rather than that of children attending a non-slum clinic in the same city. Local factors, such as crowding, may enhance the frequency of GAS transmission and horizontal gene transfers that contribute to increased strain diversity in the slums. GAS vaccine coverage and control of GAS infections will need to take these local factors and strain differences into consideration.

## Background

Group A *Streptococcus *(*Streptococcus pyogenes; *GAS) causes a wide spectrum of diseases, including pharyngitis and pyoderma to more severe diseases such as toxic shock syndrome, necrotizing fasciitis, glomerulonephritis and rheumatic heart disease (RHD) [1]. Children are the major reservoir of GAS [2]. The highest prevalence of GAS infections and their complications are found in developing countries [3].

GAS strain typing is frequently used to characterize the epidemiology and pathogenesis of GAS infections. The most common target of typing methods is the M protein, which is a cell surface virulence factor serving as a target of the immune response to GAS that confers type-specific resistance. A sequence-based typing system called *emm *sequence typing, based on the N-terminus hypervariable region (5') of the M protein gene, is now widely used [4]. Many studies have been conducted using *emm *typing to show associations of specific strain types with disease outcomes [5-9]. Information about geographic *emm *type distribution can also be used to assess candidate vaccine coverage [10,11], including that of a 26-valent vaccine that has recently completed phase II trials [12].

Epidemiologic studies have revealed that developing countries have high *emm *type diversity [13,14], while high-income countries are more likely to have a limited number of *emm *types [15-18]. This pattern was clearly demonstrated in a recent systematic review of 120 articles and reports on GAS *emm *types [19]. It found a higher diversity of *emm *types in Africa and the Pacific compared to high-income countries, which may be related to differences in geographic, environmental, socioeconomic or host factors. However, comparisons which are made across continents cannot evaluate the impact of local factors on genotype distribution. In this study, we compared the *emm *types of GAS isolates obtained from children in slum and wealthy neighborhoods in the same city, Salvador, Brazil to examine the influence of urban environmental, demographic, and socioeconomic factors on diversification of GAS. Furthermore, we collected isolates from children with and without sore throat to identify associations of certain *emm *types with clinical outcome.

## Methods

### Study Sites

This study was conducted at three pediatric outpatient emergency clinics (clinics A, B, and C). Clinical services at Clinic A and Clinic B are offered free to patients through the publicly funded Unified Health System (SUS). These clinics serve primarily low-income patients. The socioeconomic status and demographic characteristics (household density, income, education level of mother and father) of patients seeking care at Clinic A and Clinic B are similar. Clinic C serves wealthier clientele and only those with private insurance. Clinic B is located 24 km from Clinic C, Clinic A is located 32 km from Clinic B, and Clinic A is located 11 km to Clinic C. The size of the patient population is comparable between the three clinics. Each clinic receives about 5000-6000 children as outpatients each year.

### Patient Recruitment

Patients aged 3-15 years were consecutively recruited from Clinics A-C from April 17, 2008 to October 31, 2008. Recruitment occurred while patients waited for their medical evaluation, or immediately following their appointment. Parents/guardians of children were approached by a research team member for recruitment and consent to participate in the study. At this time, a brief description of the study, risks, benefits, and issues of confidentiality were provided. Following consent from parents/guardians and verbal assent from minors, a trained member of the research team administered a standardized questionnaire and collected a throat swab sample of the study participant. All members of the research team were trained in standardized technique for both procedures. Institutional Review Board (IRB) approval was obtained from all hospitals, the Comissão Nacional de Ética em Pesquisa (*Conep*) (National Bioethics Commission of Brazil), the Comitê de Ética em Pesquisa-Centro de Pesquisa Gonçalo Moniz - Fiocruz (Ethics Committee for Research - Fiocruz), and the University of California, Berkeley Committee for the Protection of Human Subjects.

#### Definitions

Slum communities were defined according to the United Nations Human Settlements Program as human settlement areas that have one or more of the following characteristics: 1) inadequate access to safe water; 2) inadequate access to sanitation and other infrastructure; 3) poor structural quality of housing; 4) overcrowding; and 5) insecure residential status [20]. In the neighborhoods served by Clinic A and B, these characteristics vary at the individual household level. However, what these slum settlements all have in common is that they were illegally built on land with unclear tenure status. The structural quality of housing also varies among residential units in these slums (red brick houses with unfinished wall and tile roof; shacks made of discarded lumber and corrugated tin roof), but the common characteristics are that they were all built with no official permit.

Cases were defined as those children whose chief complaint was sore throat. GAS culture-positive sore throat was defined as a child with a sore throat in whom GAS was isolated from the throat swab. Controls were defined as those visiting the clinics for other reasons. Exclusion criteria included use of antibiotics in the past two weeks, or any illness requiring inpatient hospitalization on the day of recruitment. Slum children were defined as those attending Clinics A and B, and non-slum children were those attending Clinic C.

### Data collection

The following variables were recorded: reason for visit to the hospital, date of birth of patient, sex of patient, household income, home address, whether in school and where, whether in daycare and where, total number of people living in house, number of children 15 years or younger in household, whether had sore throat in past six months, level of education of mother, level of education of father, and co-morbidities.

### Laboratory Sample Isolation and Identification

Swab cultures were obtained from the pharynx of the study children following a standard protocol. All study technicians were observed periodically at the clinic sites for proper and consistent swabbing technique. A sterile cotton swab tip was applied to the posterior pharynx and tonsils, as recommended by the Infectious Disease Society of America (IDSA) [21]. The swabs were immediately placed in Stuart transport medium, transported to the laboratory and plated the same day of collection on 5% sheep blood agar. The plates were incubated at 37°C for 24 - 48 h with 5% CO_2. _Streptococci were phenotypically identified by beta-hemolysis, colony morphology, and the catalase test. Carbohydrate group identification (Groups A, B, C, F, G) was performed by positive latex agglutination (Remel PathoDx Strep Grouping Latex Test Kit, Remel, Lenexa, KS, USA). Pure culture samples were stored in 5% glycerol at -80°C until further use.

#### *emm *typing

*emm*-typing of all isolates were performed as described by the Center for Disease Control and Prevention (CDC) protocol http://www.cdc.gov/ncidod/biotech/strep/protocol_emm-type.htm.

### Statistical Analysis

Analyses were conducted by STATA 11.0 (Stata Inc., College Station, Texas). Categorical variables were compared with the chi-square test. Student's t-test and ANOVA were used to compare means, and multivariable logistic regression was used to evaluate the association between specific *emm *types and case status while controlling for covariates. Models were restricted to those children with culture-positive GAS. Simpson's index of diversity was used to calculate the variation of the number of *emm *types of GAS isolates by clinic or by case status [22]. Higher index measures represent greater diversity of *emm *types, since the method calculates the probability that any two randomly selected isolates from the same population will be of different *emm *types. Confidence intervals (CI's, 95%) for the diversity index measures were calculated as previously described [23].

## Results

### Demographic and clinical characteristics

Between April 17, 2008 and October 31, 2008, 2194 children aged 3-15 years (759 in Clinic A, 518 in Clinic B, 917 in Clinic C), who met the eligibility criteria, were identified from the three study clinics. Of 2181 children with data on case status, 624 (28.6%) came with a complaint of sore throat (cases), and 1557 (71.4%) came for other illnesses. The distribution of reasons for visit among the controls was comparable across the three hospitals (data not shown).

The mean age of all the study children differed between slum (7.2 years) and non-slum residents (7.8 years)(p < 0.001, Table 1). The sex distribution did not differ between slum and non-slum populations. One minimal monthly salary (MS) or less (equivalent to US$246.10 as of April 2008) was reported by 648 (53.8%) of 1205 slum households, and 37 (4.3%) of 870 non-slum households (P < .001). Salary was positively correlated with level of education in this study. The mean number of members per household in the slum population (4.5) was greater than that in the non-slum population (4.0) (p < .001). The mean number of children <15 years per household was greater in the slum population (2.0 persons) than that in the non-slum population (1.6 persons) (p < .001).

**Table 1:** Demographic characteristics and streptococcal group distributions of children attending slum and non-slum clinics

	Total Population (n = 2194)	**Non-slum**^**a **^**(n = 917)**		**Slum**^**b **^**(n = 1277)**		
		**N**		**N**		**p-value**

**Cases**	624	915	287	1266	337	--

**Controls**	1557	915	628	1266	929	--

**Mean Age in years (95%CI)**	7.5 (7.3-7.6)	917	7.8 (7.6-8.1)	1277	7.2 (7.1-7.4)	<.001

**Sex**						1.0
**Female**	1060	917	443	1277	617	
**Male**	1134	917	474	1277	660	

**Monthly salary**		870		1205		<.001
≤**415**	685		37		648	
**416-830**	550		161		389	
**831-1660**	336		204		132	
**1661-2490**	195		173		22	
≥**2491**	309		295		14	

**Mean # people/house (95%CI)**	4.3 (4.2-4.3)	917	4.0 (3.9-4.1)	1275	4.5 (4.4-4.6)	<.001
**Mean # people **≤ **15 yrs./house (95%CI)**	1.8 (1.8-1.9)	917	1.6 (1.5-1.6)	1276	2.0 (2.0-2.1)	<.001

**Group A**	254	917	99	1277	155	0.33

**Group B**	34	917	24	1277	10	.001

**Group C**	57	917	30	1277	27	0.09

**Group F**	51	917	24	1277	27	0.44

**Group G**	133	917	46	1277	87	0.08

^a^Includes all children from Clinic C

^b^Includes all children from Clinics A and B

N: total number with available response

The difference in mean age between cases (7.4 years) and controls (7.5 years) or between patients who tested positive for GAS (7.6 years) or negative for GAS (7.5) were not significant, both in the total study population, and when stratified by slum status.

### Microbiologic studies

In total, 529 *Streptococcus *isolates (groups A-G) from 2194 children were obtained (Table 1). Of these, 254 (48%) were GAS (Table 2). Of 253 GAS isolates (1 isolate missing case/control status), 125 (8%) were from controls and 128 (20.5%) were obtained from cases (p < .001). The proportion of cases who tested culture positive for GAS differed by slum (23.1%) vs. non-slum clinic subjects (17.4%), which approached statistical significance (p = 0.08). The proportion of controls that tested positive for GAS did not differ between slum vs. non-slum children (7.8% vs. 8.2%).

**Table 2:** Demographic and streptococcal group distributions, by sore throat and carriage in slum versus non-slum children

	**Non-slum (n = 915) **^**a**^			**Slum (n = 1266) **^**b**^		
	
	**Case (%)**^**c**^	**Control (%)**^**c**^	p-value	Case (%)	Control (%)	p-value
	
	287 (31.4)	628 (68.6)		337 (26.6)	929 (73.4)	
**Mean Age (95% CI)**	7.8 (7.3-8.2)	7.9 (7.6-8.1)	0.67	7.2 (6.8-7.5)	7.3 (7.1-7.5)	0.57

**Male**	139 (48.4)	335 (53.3)	0.17	146 (43.3)	508 (54.7)	<.001

**Monthly Salary**	N = 274	N = 594	0.02	N = 312	N = 882	.75
≤**415**	11 (4.0)	26 (4.4)		178 (57.1)	468 (53.1)	
**416-830**	38 (13.9)	123 (20.7)		91 (29.2)	291 (33.0)	
**831-1660**	58 (21.2)	145 (24.4)		34 (10.9)	96 (10.9)	
**1661-2490**	55 (20.1)	117 (19.7)		5 (1.6)	17 (1.9)	
≥**2491**	112 (40.9)	183 (30.8)		4 (1.3)	10 (1.1)	

**Mean # persons per household (95% CI)**	4.0 (3.9-4.2)	4.0 (3.9-4.1)	0.96	4.5 (4.3-4.8)	4.4 (4.3-4.5)	0.27

**Mean # < 15 yrs. per household (95% CI)**	1.5 (1.5-1.6)	1.6 (1.6-1.7)	0.16	2.1 (2.0-2.3)	2.0 (1.9-2.1)	0.06

**Group A**	50 (17.4)	49 (7.8)	<.001	78 (23.1)	76 (8.2)	<.001

**Group B**	7 (2.4)	17 (2.7)	0.81	4 (1.1)	6 (0.6)	0.34

**Group C**	8 (2.8)	22 (3.5)	0.57	6 (1.8)	21 (2.3)	0.60

**Group F**	6 (2.1)	18 (2.9)	0.50	7 (2.1)	20 (2.2)	0.93

**Group G**	8 (2.8)	38 (6.1)	0.04	17 (5.0)	70 (7.5)	0.12

**No isolate**	208 (72.5)	475 (75.6)	0.17	225 (66.8)	736 (79.2)	<.001

^a ^Includes all patients from Clinic C. Case/control status missing for 2 study participants

^b ^Includes all patients from Clinics A and B. Case/control status missing for 11 study participants

^c ^Case = sore throat; control = carriage

### *emm *diversity

Of 254 GAS isolates, 238 yielded interpretable *emm *sequences. These 238 isolates represented 61 unique *emm *types. In the non-slum population, 94 isolates comprised 36 distinct *emm *types (38.3%). In the slum population, 144 isolates comprised 53 distinct *emm *types (36.8%). Between these two groups, the proportion of unique *emm *types did not differ (p = 0.81). The proportion of unique *emm *types was higher for carriage isolates than for sore throat cases in the slum population, but this finding did not reach statistical significance (p = 0.11) (Table 3).

**Table 3:** Diversity of *emm *types in non-slum versus slum populations

	Simpson's Diversity Index	CI	Number of Unique *emm *Types	Number of Isolates	Proportion Unique
**All**	0.95	(0.94-0.97)	61	238	26.1%
**Non-Slum**	0.92	(0.89-0.96)	36	94	38.3%
Case	0.90	(0.84-0.97)	24	49	49.0%
Carriage	0.93	(0.88-0.97)	21	45	46.7%
**Slum**	0.97	(0.96-0.98)	53	144	36.8%
Case	0.96	(0.94-0.98)	34	74	45.9%
Carriage	0.98	(0.97-0.99)	41	69	59.4%

Simpson's diversity index for all the *emm *types was 96% (94%-97%). The index was 92% (89%-96%) for the non-slum GAS *emm *types, and 97% (96%-98%) for slum *emm *types. The CI's for slum vs. non-slum only overlap at the lower bound estimate for slum, and the upper bound estimate of non-slum. Separating the two slum communities, the Simpson's diversity index was 96% (94%-98%) for Clinic A, and 97% (96%-99%) for Clinic B. For both slum and non-slum populations, the diversity index was lower for cases than for controls [non-slum: 90% vs. 93%; slum 96% vs. 98%] (Table 3).

**Figure 1:**
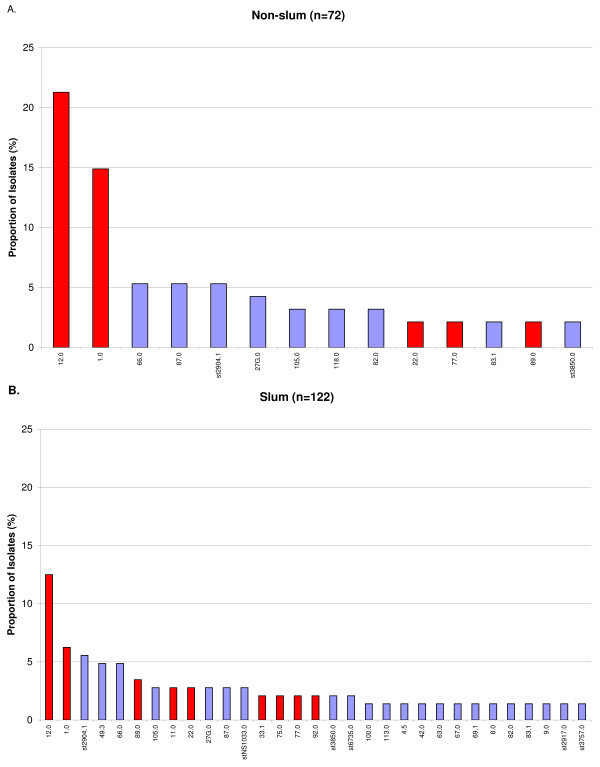
**Proportion of the most common *emm *types in non-slum (A) and slum (B) populations**. Only *emm *types represented by more than one isolate are included in the graphs. Bars in red indicate *emm *types included in the 26-valent vaccine. Blue bars indicate *emm *types not included in the vaccine.

In both slum and non-slum populations, *emm*12.0 was the predominant type, followed by *emm*1.0 (Figure 1). However, in the non-slum population, 20 (21.3%) of 94 isolates were *emm*12.0, whereas in the slum population, 18 (12.5%) of 144 isolates were *emm*12.0 (p = 0.07). *emm*1.0 was the second most prevalent *emm *type in both populations, and also constituted a larger proportion of non-slum isolates. In the non-slum population, 14 (14.9%) of 94 isolates were *emm*1.0, and in the slum population, *emm*1.0 constituted 9 (6.3%) of 144 isolates (p = 0.03). The three most predominant *emm *types constituted a much larger proportion of isolates in the non-slum population as compared with the slum population (41.5% vs. 24.3%)(p = 0.005), suggesting that GAS diversification was greater in the slum population.

Only 7 *emm *types 12.0 (n = 38), 1.0 (n = 23), st2904.1 (n = 13), 66.0 (n = 12), 87.0 (n = 9), 49.3 (n = 8), and 27G.0 (n = 8) comprised 46.6% of the total. There were 22 *emm *types that were represented by only a single isolate. In total, 25 distinct *emm *types were detected only in slum children, compared with only 8 *emm *types which were found only in non-slum children. *emm *types which were represented by only one isolate were more likely to be found in children 10 years or older, than in those 9 and younger, which approached statistical significance (p = 0.08).

#### *emm *type and case status

Three *emm *types were significantly associated, conferring either risk or protection, with sore throat. Of those who tested positive for GAS, those with *emm*12.0 (n = 38) had 2.2 times the odds of having sore throat compared with those with a different *emm *type (p = 0.04), after adjusting for age, income and number of children less than 15 years of age in the household (Table 4). For those patients with *emm*66.0 (n = 12), the odds of sore throat were 8.7 times that of sore throat with other *emm *types in the multivariable model (p = 0.04). Interestingly, an inverse relationship was seen for *emm*27G.0 (n = 8), where those with this *emm *type had 0.1 times the odds of sore throat compared with other *emm *types in the multivariable model (p = 0.07).

**Table 4:** Common *emm *types and association with sore throat or carriage, among GAS culture-positive (n = 253) patients

*Emm *Type	N (%)	Case (n = 128)	Control (n = 125)	Crude OR	p-value	95% CI	**OR **^**a**^	p-value	95% CI	**Multivariable OR**^**b**^	p-value	95% CI
**12.0**	38 (15.0)	25	13	2.09	0.05	1.0-4.3	2.12	0.04	1.0-4.4	2.21	0.04	1.1-4.7
**1.0**	23 (9.1)	11	12	0.89	0.78	.4-2.1	0.88	0.77	.4-2.1	0.84	0.69	.4-2.0
**st2904.1**	13 (5.1)	7	6	1.15	0.81	.4-3.5	1.15	0.80	.4-3.5	1.13	0.83	.4-3.5
**66.0**	12 (4.7)	9	2	4.65	0.05	1.0-22.0	4.64	0.05	1.0-21.9	8.70	0.04	1.1-70.5
**87.0**	9 (3.6)	5	4	1.23	0.76	.3-4.7	1.22	0.77	.4-7.1	1.29	0.71	.3-5.0
**49.3**	8 (3.2)	5	3	1.65	0.5	.4-7.1	1.66	0.50	.4-7.1	1.13	0.88	.2-5.8
**27G.0**	8 (3.2)	1	7	0.13	0.06	.0-1.1	0.13	0.06	.0-1.1	0.14	0.07	.0-1.1
**Unique**	22 (8.7)	10	12	0.80	0.61	.3-1.9	0.79	0.61	.3-1.9	0.65	0.39	.3-1.7

^**a**^Adjusted for age

^**b**^Adjusted for age, income, number <15 yrs. in household

#### Vaccine Coverage

In this study, 100 (42.0%) of the 238 *emm *typed isolates, and 15 (24.6%) of the 61 *emm *types would be covered by the 26-valent M-protein-based GAS vaccine, assuming cross-immunity between type 1.2 and subtype 1.25, between type 101 and subtype 101.1, and between type 33.0 and subtype 33.1 [12]. Stratifying by populations, 45 (47.9%) of 94 isolates and 10 (27.8%) of 36 *emm *types from non-slum children would be covered by the vaccine. In the slum, the coverage for isolates would be 52 (36.1%) of 144 isolates, and 11 (20.8%) of 53 *emm *types.

In children presenting with sore throat, 55 (44.7%) of 123 *emm *typed isolates, and 13 (30.2%) of 43 *emm *types would be covered by the current 26-valent M-protein-based GAS vaccine. In the non-slum population, 25 (51.0%) of 49 *emm *typed isolates, and 8 (33.3%) of 24 *emm *types would be covered. In the slum population, 32 (43.2%) of 74 *emm *typed isolates, and 10 (29.4%) of 34 *emm *types would be covered by the vaccine.

## Discussion

In Salvador, Brazil we found significant differences in *emm *type diversity among GAS isolates obtained from different populations in the same city. The diversity index was significantly higher among GAS isolates from children residing in slum communities (97%) compared to those living in wealthier neighborhoods (92%). In fact, the diversity index of the non-slum GAS isolates was closer to that of *emm *types reported from high income countries (92%) than to those found in the slum populations of the same city Salvador [19]. This study suggests that GAS strain diversification may be influenced by local factors. Such factors may include crowding which is more prevalent in slum communities. Crowding may facilitate increased transmission opportunities and possible horizontal gene transfers that contribute to strain diversification. For socioeconomic reasons, slum residents are also less likely to undergo antibiotic treatment for sore throat. Our data found significant differences in household density, type of health insurance plan, and income between slum versus non-slum communities (Table 1).

In addition to *emm *type differences in GAS strains across high-income vs. low-income populations in the same city, we found certain *emm *types to be over or under-represented among children with sore throat [*emm*66.0 (OR = 8.7), *emm*12.0 (OR = 2.2), *emm*27G.0 (OR = 0.1)]. Furthermore, we were able to identify clinically relevant strains that comprised less than 5% (emm66.0) and less than 3% (emm27G.0) of the sample population. Further laboratory studies are warranted to determine why certain *emm *types predominate in clinical cases (emm66.0, emm12.0) or are inversely associated with sore throat (emm27G.0).

A vaccine against GAS will have substantial benefits worldwide. However, the impact on disease reduction could vary by region depending on the vaccine composition. Currently, the only vaccine to complete phase I/II trials is a 26-valent recombinant M protein vaccine [12,24]. In our study, only 42% of the total isolates, and 44.7% of isolates from cases, would be covered by the 26-valent M-protein-based GAS vaccine. Furthermore, the coverage of the 26-valent vaccine in all slum (36%) versus non-slum (48%) isolates would not be equal even within the same city.

## Conclusions

This study suggests that local demographic and socioeconomic factors may contribute to the diversification of GAS *emm *types, and that distinct bacterial population distribution occurs between different neighborhoods separated by 11-32 km in the same city. This distinction might be particularly pronounced in cities with slums. We note that it is not simply poverty itself that determines this difference [25,26]. As the world expands toward the projected population size of two billion slum residents in less than 30 years, it will be essential to better elucidate the slum structural dynamics which contribute to major differences in disease outcomes and vaccine coverage [20].

## Financial support

Centers for Disease Control and Prevention Grants for Public Health Research Dissertation (R36).

## Potential conflicts of interest

The authors declare that they have no competing interests.

## Authors' contributions

ST carried out the sample collection, genetic strain typing, statistical analysis, and drafted the manuscript. JR participated in the design of the study, the laboratory coordination and protocol design, and edited the manuscript. AA participated in the design and coordination of the study and helped to draft the manuscript. RR contributed to study design and coordination and helped to draft the manuscript. MR contributed to the design and coordination of the study and helped to draft the manuscript. LR contributed to the design, study coordination, statistical analysis, and helped to draft the manuscript. All authors read and approved the final manuscript.

## Pre-publication history

The pre-publication history for this paper can be accessed here:

http://www.biomedcentral.com/1471-2334/10/327/prepub
